# Investigating Endocrine Causes in Fluid Non-responsive Hypotension: An Interesting Case of a Pituitary Macroadenoma

**DOI:** 10.7759/cureus.75843

**Published:** 2024-12-16

**Authors:** Shweta S Acharya, Ashok Kumar, Ashok Kumar

**Affiliations:** 1 Internal Medicine, Max Smart Super Speciality Hospital, New Delhi, IND

**Keywords:** adrenal insufficiency, corticosteroids, euvolemia, fluid non-responsive, hypotension, pituitary macroadenoma, secondary hypothyroidism

## Abstract

In this case, the message is conveyed that after ruling out sinister causes of hypotension, endocrine causes should also be considered, particularly in the light of a relatively long history, absence of any sepsis and organ dysfunction, preserved urine output, euvolemic status, and with no significant response to intravenous fluid. In our case, a patient with hypotension with relatively stable other clinical parameters has been evaluated to reveal pituitary macroadenoma as an underlying diagnosis.

## Introduction

Hypotension is a medical emergency that requires prompt attention and management to identify and address its underlying cause. Various factors can lead to hypotension, including sepsis, fluid or blood loss, drug-induced effects, cardiac and rhythm disorders, and endocrine causes [1]. This case highlights the importance of considering endocrine etiologies of hypotension. As reviewed by Vantyghem et al., chronic hypotension may result from endocrine disorders such as adrenal failure (primary or secondary), hypoaldosteronism, diabetic dysautonomia, or carcinoid syndrome [2].

We present a case of a woman in her late 30s with hypotension unresponsive to intravenous fluids. The patient was euvolemic with preserved urine output. Subsequent investigations revealed a pituitary macroadenoma causing secondary adrenal failure. Following neurosurgical intervention via sublabial transsphenoidal tumor debulking, her blood pressure and hormonal parameters significantly improved.

## Case presentation

A woman in her late 30s presented to the emergency room with generalized weakness, nausea, and poor oral intake for a month. Over the preceding 15 days, she experienced excessive lethargy and sleepiness. She reported no fever, cough, breathlessness, lumps in the axilla, neck, or groin, or visual complaints. She also denied bowel or bladder symptoms, recurrent infections, or decreased urine output.

Her medical history was unremarkable, with no history of diabetes, hypertension, or thyroid disorders. She had not been on long-term medications. Her obstetric history included a single live birth four years ago, and she has been amenorrheic for the past two months.

On examination, the patient appeared thinly built with a body mass index of 18 kg/m². Vital signs showed sinus tachycardia and hypotension (blood pressure: 88/50 mmHg). General and systemic examinations were unremarkable, except for bilateral temporal visual field loss on bedside neurological testing. Pupils were reactive to light bilaterally. Cardiovascular, respiratory, and abdominal examinations showed no abnormalities beyond hypotension and tachycardia.

Investigations

**Figure 1:**
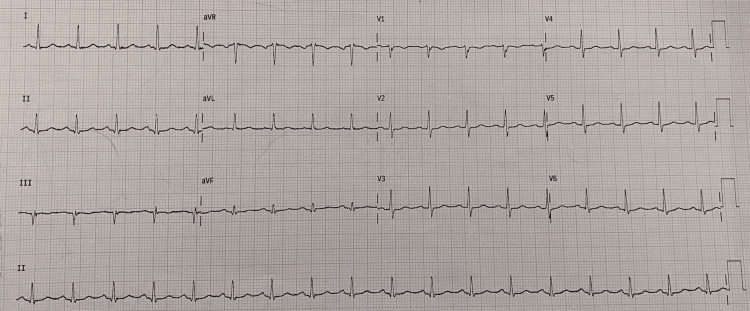
ECG showing sinus tachycardia. There is no evidence of any chamber enlargement, rhythm abnormality, or evidence of coronary artery disease to contribute to hypotension ECG: electrocardiogram

The initial workup included an electrocardiogram (ECG) (Figure 1) showing sinus tachycardia and a 2D echocardiogram within normal limits. Despite 1 liter of fluid resuscitation, her blood pressure remained at 88/50 mmHg, although urine output was normal. Infectious causes were ruled out with a normal total leukocyte count, urine microscopy, and a chest X-ray (Table 1).

**Table 1:** List of investigations HbA1c: glycosylated hemoglobin, ALT: alanine aminotransferase, AST: aspartate aminotransferase, TSH: thyroid-stimulating hormone, FT3: free tri-iodothyronine, FT4: free tetra-iodothyronine, beta hCG: beta human chorionic gonadotropin, FSH: follicle-stimulating hormone, LH: luteinizing hormone, ACTH: adrenocorticotropic hormone, MRI: magnetic resonance imaging

Test	Patient's value	Reference ranges
Hemoglobin	12.9 gm/dL	12-15 gm/dL
Creatinine	0.5 mg/dL	0.5-1.04 mg/dL
Serum sodium	128 mmol/L	136-146 mmol/L
Serum potassium	4.0 mmol/L	3.5-5.1 mmol/L
Spot urine sodium	52 mmol/L	<20 mmol/L
Serum bicarbonate	23 mmol/L	22- 26 mmol/L
Serum osmolality	283 mOsm/kg H2O	282-303 mOsm/kg H2O
HbA1c	4.89%	4-6%
ALT	25 IU/L	13-41 IU/L
AST	11 IU/L	14-54 IU/L
TSH	0.31 µIL/mL	0.34-5.6 µIL/mL
FT3	2.34 pg/mL	2.5-3.9 pg/mL
FT4	0.49 ng/dL	0.58-1.64 ng/dL
Serum growth hormone	0.45 ng/mL	0.010-3.607 ng/mL
Serum beta hCG	<0.50 mIU/mL	0-5.0 mIU/mL
CA 19.9	12.16 U/mL	0-35 U/mL
CA 125	10.80 U/mL	0-35 U/mL
Serum FSH	5.25 mIU/mL	Midcycle = 4.54-22.51 mIU/mL; follicular phase = 3.85-8.78 mIU/mL; postmenopausal = 16.74-113.59 mIU/mL
Serum LH	0.20 mIU/mL	Midcycle = 19.18-103.03 mIU/mL; luteinizing hormone = 1.2-12.86 mIU/mL; follicular phase = 2.12-10.89 mIU/mL; postmenopausal = 10.87-58.64 mIU/mL
Serum prolactin	240 ng/mL	Pre-menopausal = 3.34-26.74 ng/mL; postmenopausal = 2.74 - 19.64 ng/mL
25-Hydroxy vitamin D	34.15 ng/mL	30-100 ng/mL
Serum morning cortisol	4.08 µg/dL	6.7-22.6 µg/dL
Serum morning ACTH	6.2 pg/mL	9-52 pg/mL
MRI brain with sella, contrast	A large, solid, heterogeneous sellar-suprasellar mass lesion is seen with a "figure of 8" appearance, measuring approximately 43 x 34 x 32 mm (craniocaudal x transverse x anteroposterior). The pituitary gland is not visualized separately. The lesion abuts the bilateral internal carotid arteries (Knosp classification of cavernous sinus invasion by pituitary macroadenomas: Grade 2 on the right and Grade 3A on the left). Superiorly, it extends up to the floor of the third ventricle, compressing and indenting it, with consequent mild dilatation of the bilateral lateral ventricles and periventricular hyperintensities suggestive of possible ooze. Posteriorly, the lesion is splaying the cerebral peduncles, while anteriorly, it is indenting the fornix and bilateral anterior cerebral arteries, which are draped over it. The lesion causes superior displacement of the optic nerves and complete effacement of the optic chiasma. On imaging, the lesion shows heterogeneous signals: a mixed hypointense and hyperintense signal on T2-weighted imaging and hypointense signals on T1-weighted imaging, with obvious evidence of bleeding or blooming.	

Given her two-month history of amenorrhea, obstetric and gynecological consultations ruled out pregnancy based on low beta human chorionic gonadotropin levels and normal abdominal and pelvic ultrasounds. Anemia was excluded (hemoglobin: 12.9 g/dL), and other laboratory tests showed normal liver function and serum creatinine levels. However, serum sodium was low (128 mg/dL), urinary spot sodium was elevated, and serum osmolality was within the normal range.

Blood samples were sent for endocrine evaluation, and while reports were awaited, she was started on corticosteroids. A subsequent endocrine evaluation revealed low morning serum cortisol levels, low adrenocorticotropic hormone (ACTH), low thyroid-stimulating hormone (TSH), and low free triiodothyronine (T3) and thyroxine (T4) levels. Serum follicle-stimulating hormone (FSH) and luteinizing hormone (LH) levels were also low, while serum prolactin was elevated. Random blood sugar was 80 mg/dL, and glycosylated hemoglobin was 4.89%. Ovarian tumor markers (CA-125 and CA 19.9) were not raised.

Imaging and ophthalmological findings

**Figure 2:**
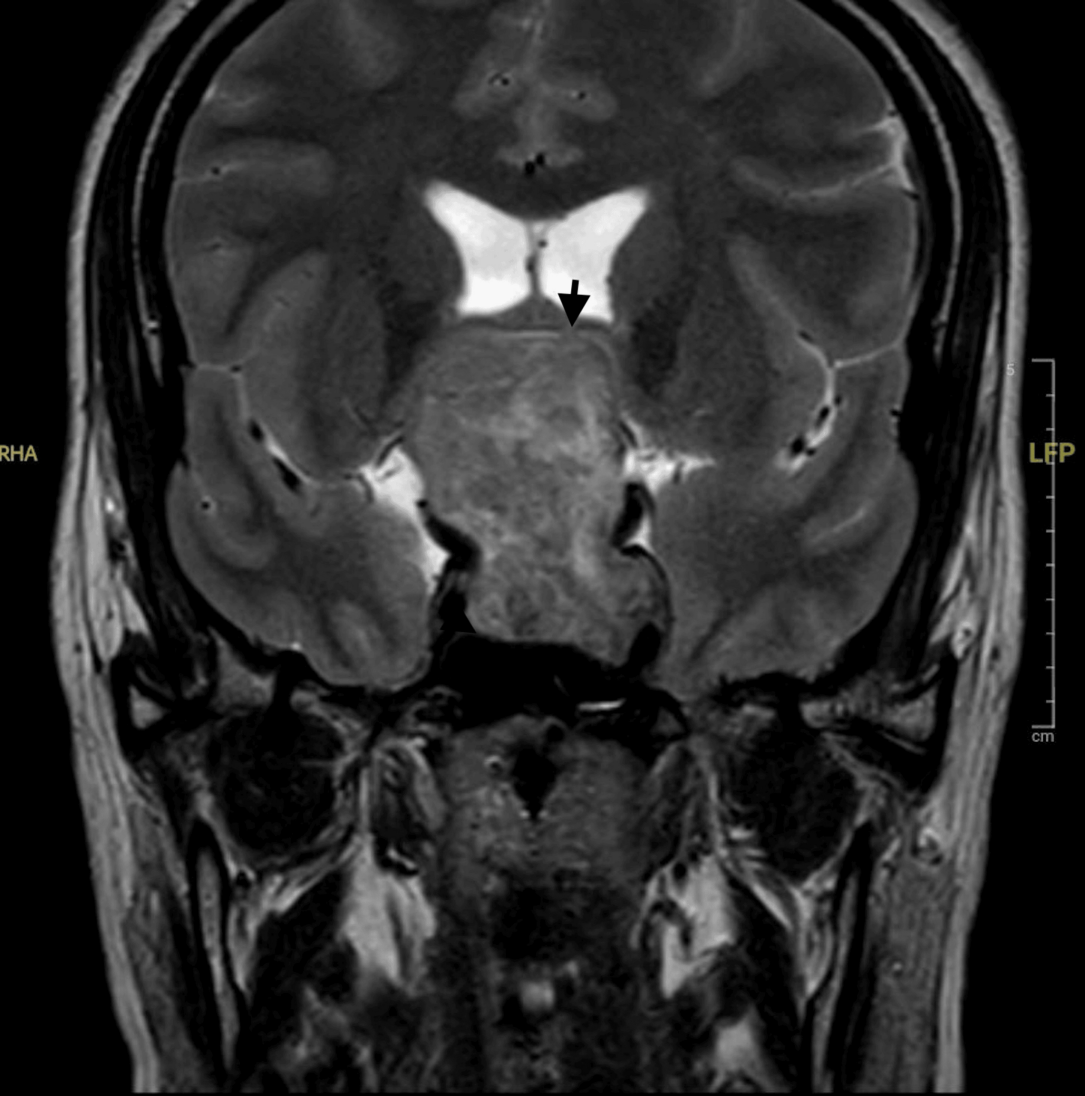
T2-weighted coronal section of the brain showing a large solid (black arrow), heterogenous sellar, and suprasellar mass lesion mixed hypo and intense signals. The pituitary gland is not seen separately

**Figure 3:**
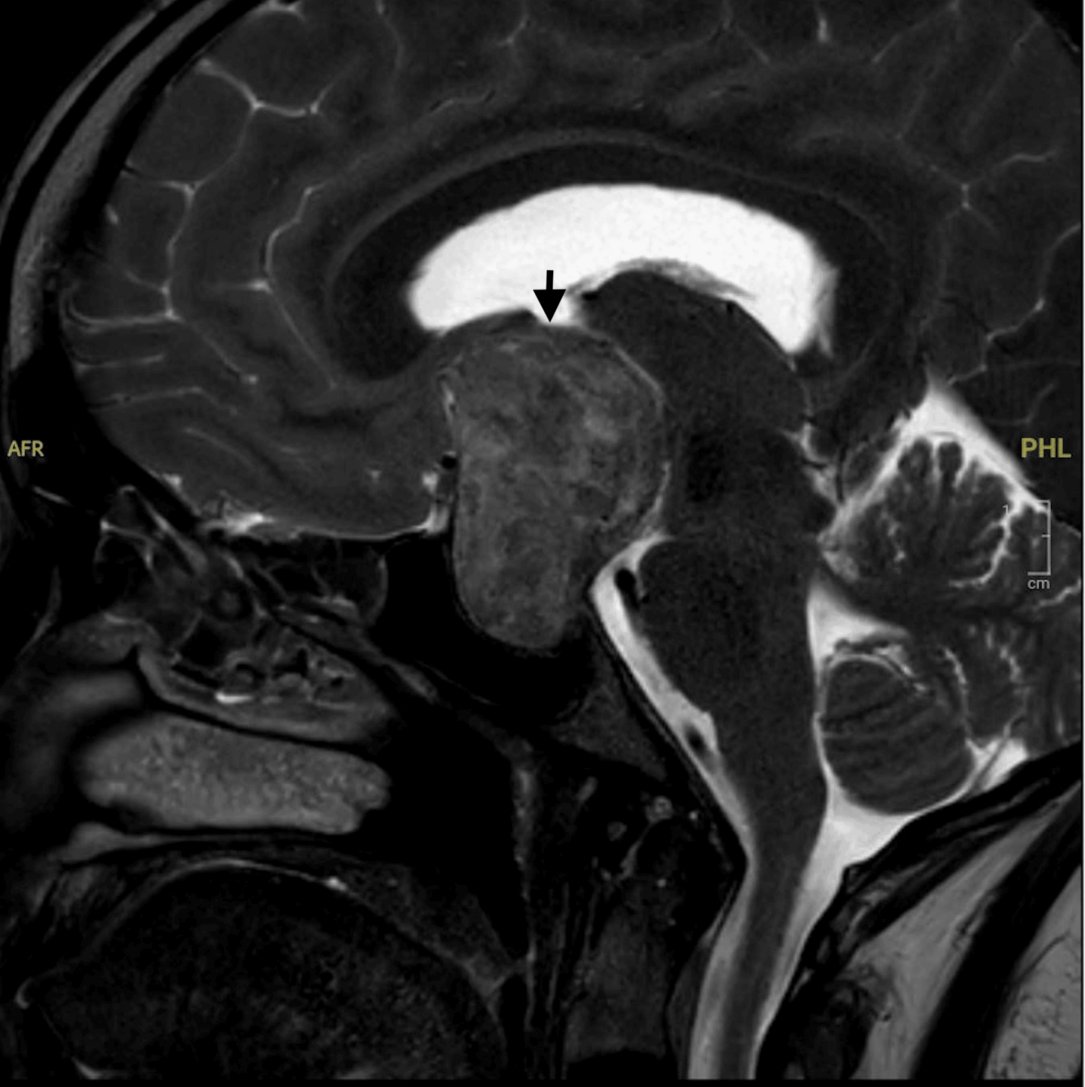
T2-weighted sagittal section of the brain showing a large solid, heterogenous sellar suprasellar mass lesion (black arrow) measuring about 34 x 32 mm (transverse and anteroposterior). The pituitary gland is not seen separately

**Figure 4:**
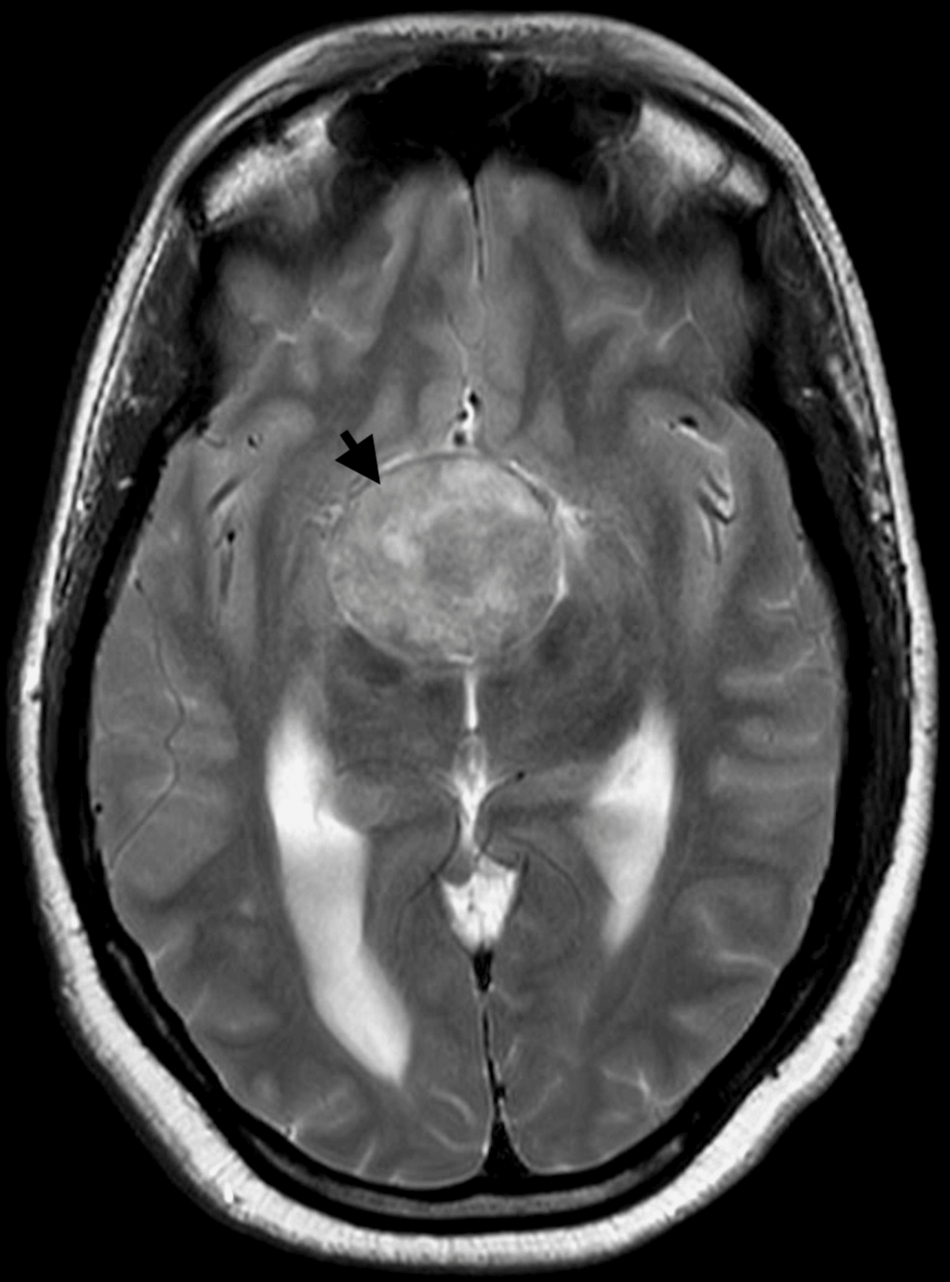
T2-weighted transverse sections: The lesion (black arrow) abuts bilateral internal carotid arteries (Knosp classification of cavernous sinus invasion by pituitary macroadenomas—Grade 2 on the right and grade 3A on the left side). It is reaching superiorly up to the floor of the third ventricle, compressing it, with consequent mild dilatation of bilateral lateral ventricles with periventricular hyperintensities. Posteriorly, it is splaying cerebral peduncles. Anteriorly indenting the fornix and bilateral anterior carotid arteries, which are draped over it. The lesion causes superior displacement of the optic nerves and complete effacement of the optic chiasma. Pituitary macroadenoma marked with a black arrowhead

**Figure 5:**
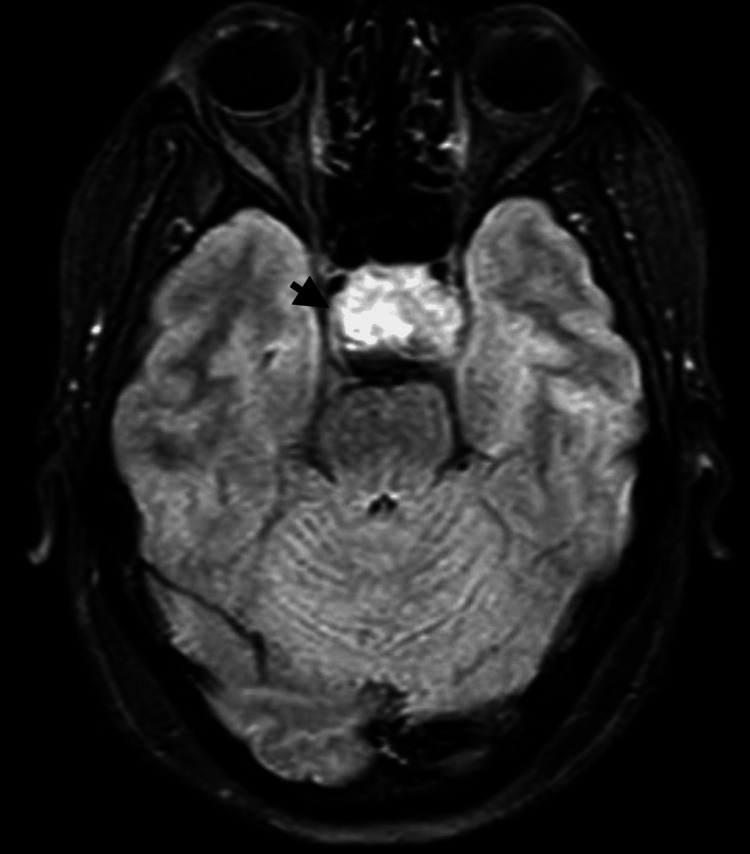
T2 flair transverse section of the brain showed a large solid, heterogenous sellar suprasellar mass lesion with mixed hypo and intense signals (black arrow). Suggestive of pituitary macroadenoma

MRI of the brain revealed a large sellar and suprasellar mass consistent with a pituitary macroadenoma. The mass compressed the optic chiasm and third ventricle, causing mild dilation of the lateral ventricles and periventricular ooze. A contrast-enhanced MRI confirmed these findings and provided additional details of tumor margins (Figures 2-5).

Ophthalmological examination of distant vision in the right eye, as per Snellen's chart, was 6/12, and in the left eye was 6/6. Intraocular pressure was normal bilaterally, and fundus examination revealed no papilledema. Perimetry confirmed bitemporal hemianopia.

Management and outcome

The patient was started on corticosteroids, thyroid hormone replacement, cabergoline, medroxyprogesterone acetate for secondary amenorrhea, and fluid restriction. Following pre-anesthetic clearance, neurosurgical intervention was performed in the form of sublabial transsphenoidal tumor debulking.

The intraoperative and postoperative periods were uneventful. The patient showed significant symptomatic improvement, with blood pressure normalization and restoration of vision to 6/6 bilaterally. At a one-month follow-up, repeat hormone assays demonstrated normal TSH, prolactin, and morning serum cortisol levels, along with normalized serum sodium.

## Discussion

Pituitary tumors arise predominantly from the anterior pituitary and are, in the majority of cases, benign, with a slow growth rate. Tumors larger than 10 mm are classified as macroadenomas, while those smaller than 10 mm are termed microadenomas. Functioning pituitary adenomas secrete one or more anterior pituitary hormones, whereas non-functioning adenomas cause hormone deficiencies by compressing surrounding structures. Thus, a multidisciplinary approach involving a neurosurgeon, an endocrinologist, and an opthalmologist is recommended [3-5]. Common mass effect features include headaches and visual impairment, such as bitemporal hemianopia [6,7]. Deficiencies in anterior pituitary hormones, including gonadotropins (FSH and LH), growth hormone, TSH, and ACTH, may occur singly or in combination, along with prolactin excess due to pituitary stalk compression. Patients with secondary hypothyroidism often show low TSH and FT3/FT4 levels.

In our patient, persistent hypotension that was unresponsive to fluids combined with euvolemia and normal urine output raised suspicion for an endocrine cause. Hormonal assays revealed secondary adrenal insufficiency, secondary hypothyroidism, and hyperprolactinemia. MRI findings confirmed the presence of a pituitary macroadenoma, causing compression of the optic chiasm and third ventricle. The absence of hyperkalemia and metabolic acidosis ruled out primary adrenal insufficiency, while the lack of intermittent flushing or respiratory distress excluded carcinoid syndrome. Orthostatic hypotension was also ruled out as the patient’s hypotension persisted in the supine position.

Secondary adrenal insufficiency results from reduced ACTH secretion, leading to cortisol deficiency. Importantly, mineralocorticoid function remains intact due to regulation by the renin-angiotensin system, independent of hypothalamic-pituitary signaling. Chronic adrenal insufficiency typically presents with an insidious onset, whereas acute adrenal insufficiency is critical and often life-threatening [8-12]. Cortisol deficiency affects autonomic cardiovascular regulation, enhancing arterial baroreflex sensitivity and lowering baseline blood pressure [13-16]. Furthermore, impaired catecholamine release reduces sympathetic activity, exacerbating hypotension [17]. These mechanisms explain the persistent hypotension observed in our patient despite normal fluid status and cardiac function.

Literature on endocrine causes of hypotension is limited. Vantyghem et al. highlighted chronic hypotension as a feature of adrenal failure, whether primary or secondary, as well as hypoaldosteronism, diabetic dysautonomia, and carcinoid syndrome [2]. Our case adds to this body of evidence, emphasizing the importance of considering endocrine causes, particularly when common causes such as sepsis, cardiac dysfunction, and hypovolemia are excluded.

Treatment involves transsphenoidal resection of pituitary macroadenoma in view of bitemporal hemianopia due to optic chiasmal compression, compression of other surrounding structures, and multiple hormone deficiencies [18]. Hormonal deficiencies in our patient were managed with corticosteroids, thyroxine supplementation, and progesterone replacement for secondary amenorrhea. Dopamine agonists have been recommended as the first line in the treatment of hyperprolactinemia [19]. Our patient was, therefore, given cabergoline for managing hyperprolactinemia. Fluid restriction was also implemented to correct hyponatremia. At follow-up after one month, the patient showed normalization of blood pressure and hormonal levels, including TSH, prolactin, and morning serum cortisol.

Complications of pituitary macroadenoma include pressure effects causing compression of optic chiasma, leading to bitemporal hemianopia, pituitary hormone deficiencies, and pituitary apoplexy. Pituitary apoplexy can occur in 2-12% of cases of non-functioning pituitary adenoma and is characterized by sudden-onset severe headache. Large apoplexy may also lead to altered sensorium, signs of meningeal irritation, oculomotor nerve palsy, and bitemporal hemianopia. Management includes emergency decompression surgery and corticosteroid treatment [20]. In our patient, no evidence of pituitary apoplexy was noted.

## Conclusions

Patients presenting with hypotension require a thorough evaluation of vital signs, hydration status, and systemic function. Assessment should include a focused history and laboratory investigations to rule out common causes such as sepsis, cardiac dysfunction, or hypovolemia. When hypotension persists despite these measures, endocrine causes such as adrenal failure, whether primary or secondary, as well as hypoaldosteronism and prolactinoma should be considered, particularly in the presence of euvolemia, normal urine output, and no systemic derangement.
